# Bronchospasm During Induction of Anesthesia: A Case Report and Literature Review

**DOI:** 10.22086/gmj.v0i0.846

**Published:** 2018-05-19

**Authors:** Salman Vojdani

**Affiliations:** ^1^Anesthesia department, Fasa University of Medical Sciences, Fasa, Iran; ^2^Noncommunicable Diseases Research Center, Fasa University of Medical Sciences, Fasa, Iran

**Keywords:** Bronchial Spasm, General Anesthesia, Respiratory Hypersensitivity

## Abstract

**Background:**

Bronchospasm (spasm of bronchial muscles) in general anesthesia caused by many reasons. Untreated bronchospasm can cause hypoxia, hypotension and increased morbidity and mortality.

**Case report:**

A 28 years old female scheduled for tonsillectomy surgery. Immediately after induction of anesthesia patient developed with drop in oxygen saturation and difficulty in mechanical ventilation.

**Conclusions:**

Bronchospasm should be considered in differential diagnosis of oxygen saturation drop during general anesthesia. This situation is more common in patients without specific respiratory disorder. Tracheal irritants like sputum and blood can cause bronchospasm. Other causes include histamine or serotonin release.

## Introduction

Bronchospasm like asthma is a feature of reactive airway disease. Patients shows hyperreactive airway responses to irritants. Constriction of bronchial smooth muscle, plugging are pathophysiology of these groups.

In general anesthesia the incidence of bronchospasm in controlled asthma is approximately 2% and overall incidence is 0.2% [1]. Many patients with bronchospasm in general anesthesia had no history of airway disease. The manifestations of bronchospasm in general anesthesia are expiratory wheeze, drop in oxygen saturation and difficulty in mechanical ventilation, however in severe cases wheeze may be absent.

## Case Presentation

A 28-year,60 Kg female, American Society of Anesthesiologists(ASA) Class I was scheduled for tonsillectomy surgery. She had no history of previous surgery or any medical disorder. Chest examination was normal in preoperative anesthesia visit. Anesthesia induced with intravenous midazolam (3 mg), fentanyl (150 μg), propofol (180 mg) and atracurium (35 mg). Nasotracheal intubation performed with mild resistance. Amounts of blood was seen in throat during intubation which suctioning was done. After Nasotracheal intubation chest examination showed lack of breathing sounds. Bag ventilation was difficult to perform. Capnogram revealed prolonged expiratory upstroke, so this was suspected to bronchospasm. Oxygen saturation dropped rapidly, (Spo_2_:55%) followed by arterial hypotension (from 135/85 to 55/25 mmHg) with a moderate tachycardia (110 beats in minute) in 6 minutes after bronchial spasm. Three intravenous boluses of 100 μg epinephrine administered to correct cardiovascular instability. At the same time suctioning of the airway removed bloody secretions from trachea, then ventilation became easier and audible wheezing over both lung fields was auscultated. By giving intravenous hydrocortisone (200 mg) and aminophylline infusion (250 mg) respiratory symptoms resolved within 30 minutes. Surgery was done with uneventful clinical outcome and in the following day patient was discharged home.

## Discussion

In a study bronchospasm diagnosed 1.7 times during 1000 anesthesia [1]. Bronchospasm during perioperative period may have multiple causes from IgE-mediated anaphylaxis, non-allergic mechanism triggered by mechanical factors (i.e., orotracheal intubation), drug-induced (i.e., atracurium, morphine and meperidine) to bronchospasm in patients with hyper-reactive airway [2].

Light anesthesia, airway secretions and obstruction of the tube or circuit are other differential diagnosis. Endobronchial intubation present with unilateral wheezing [3].

If clinical symptoms persist despite appropriate therapy, it may due to pneumothorax or Pulmonary edema [2].

In cardiovascular system, the results of progressive acute bronchoconstriction are reduction of airflow, lung inflation, increased resistance of pulmonary vessels (PVR) and overload of right ventricle [3]. Propofol with bronchodilating effects considered in allergic patients, histamine-induced contractions in airway smooth muscles reduced with propofol in both healthy and asthmatic patients. Soybean oil and yolk lecithin in propofol formulation induced allergic reaction and should be used with caution in atopic patients.

In studies about allergic reactions to anesthetic agents the dominant majority were female patients, such as our study [4-6].

Other drugs with histamine release effects are neuromuscular-blocking drugs (NMBDs), intraoperative anaphylaxis often triggered by NMBDs. A few published cases reported bronchospasm triggered after spinal anesthesia. There were no intraoperative deaths [7, 5]. Since in our study Nasotracheal intubation was forceful, causes of bronchospasm may be either blood in bronchial tree or allergic reaction to anesthetic agents.

## Conclusion

One of the life threatening emergencies during general anesthesia is severe bronchospasm. In perioperative visit most of the patients had no history of allergy or asthma. Triggering factors like sputum, blood and some anesthetic agents can cause bronchospasm [1]. Selective β2 agonists (Salbutamol) are the drug of choice for treatment. The pharmacodynamics of salbutamol are poorly characterized [8]. Second-line agents include Ipratropium bromide, Magnesium sulphate, Hydrocortisone, Ketamine and in extremis Epinephrine (Nebulized and Intravenous) Extubation under deep anesthesia reduces reflex bronchoconstriction during emergence in this patients [9].

## Conflict of Interest

No conflict of interest
